# Assessing the Accuracy, Completeness, and Reliability of Artificial Intelligence-Generated Responses in Dentistry: A Pilot Study Evaluating the ChatGPT Model

**DOI:** 10.7759/cureus.65658

**Published:** 2024-07-29

**Authors:** Kelly F Molena, Ana P Macedo, Anum Ijaz, Fabrício K Carvalho, Maria Julia D Gallo, Francisco Wanderley Garcia de Paula e Silva, Andiara de Rossi, Luis A Mezzomo, Leda Regina F Mugayar, Alexandra M Queiroz

**Affiliations:** 1 Department of Pediatric Dentistry, School of Dentistry of Ribeirão Preto at University of São Paulo, Ribeirão Preto, BRA; 2 Department of Dental Materials and Prosthesis, School of Dentistry of Ribeirão Preto at University of São Paulo, Ribeirão Preto, BRA; 3 Department of Public Health, University of Illinois Chicago at College of Dentistry, Chicago, USA; 4 Department of Pediatric Dentistry, School of Dentistry of Ribeirão Preto at University of São Paulo, Ribeirão Preto, USA; 5 Department of Dentistry, School of Dentistry of Ribeirão Preto at University of São Paulo, São Paulo, BRA; 6 Department of Restorative Dentistry, University of Illinois Chicago at College of Dentistry, Chicago, USA; 7 Department of Pediatric Dentistry, University of Illinois Chicago College of Dentistry, Chicago, USA

**Keywords:** decision-making process, evidence base practice, knowledge acquisition, ai and machine learning, chat-gpt, decision-support tools, artificial intelligence in dentistry

## Abstract

Background: Artificial intelligence (AI) can be a tool in the diagnosis and acquisition of knowledge, particularly in dentistry, sparking debates on its application in clinical decision-making.

Objective: This study aims to evaluate the accuracy, completeness, and reliability of the responses generated by Chatbot Generative Pre-Trained Transformer (ChatGPT) 3.5 in dentistry using expert-formulated questions.

Materials and methods: Experts were invited to create three questions, answers, and respective references according to specialized fields of activity. The Likert scale was used to evaluate agreement levels between experts and ChatGPT responses. Statistical analysis compared descriptive and binary question groups in terms of accuracy and completeness. Questions with low accuracy underwent re-evaluation, and subsequent responses were compared for improvement. The Wilcoxon test was utilized (α = 0.05).

Results: Ten experts across six dental specialties generated 30 binary and descriptive dental questions and references. The accuracy score had a median of 5.50 and a mean of 4.17. For completeness, the median was 2.00 and the mean was 2.07. No difference was observed between descriptive and binary responses for accuracy and completeness. However, re-evaluated responses showed a significant improvement with a significant difference in accuracy (median 5.50 vs. 6.00; mean 4.17 vs. 4.80; p=0.042) and completeness (median 2.0 vs. 2.0; mean 2.07 vs. 2.30; p=0.011). References were more incorrect than correct, with no differences between descriptive and binary questions.

Conclusions: ChatGPT initially demonstrated good accuracy and completeness, which was further improved with machine learning (ML) over time. However, some inaccurate answers and references persisted. Human critical discernment continues to be essential to facing complex clinical cases and advancing theoretical knowledge and evidence-based practice.

## Introduction

Artificial intelligence (AI) is the theory and development of computer systems that can perform tasks that would normally require human intelligence [1]. The area of dentistry has increasingly adopted AI to improve the effectiveness and efficiency of dental treatments, reducing costs and treatment time [2]. The advantage of AI in dentistry is the possibility of analyzing large volumes of data, such as X-ray and tomography images, to assist in the development of diagnosis and treatment plans [2]. Furthermore, AI can help identify dental and facial pathologies, such as fractures, cavities, and periodontal diseases, with greater speed and accuracy [3-5].

The Chatbot Generative Pre-Trained Transformer (ChatGPT), developed by OpenAI in 2022, is designed for natural language processing tasks and is capable of understanding and generating human-like text based on input [6]. Trained on diverse internet text, ChatGPT finds applications in answering questions, generating text, and engaging in conversations [6]. These applications may contribute to the clinical education of dental students [7,8]. It could help them to learn, understand, and improve scientific writing for dental researchers [9-12]. 

Among the concerns surrounding using ChatGPT as a knowledge acquisition tool is its ability to provide accurate responses to questions [13,14]. Furthermore, these responses may be vague and incomplete [15], diminishing the capacity for comprehensive knowledge acquisition. Also, little is known about the accuracy and completeness of the answers generated by ChatGPT in dentistry. In the field of healthcare, this can impact the overall health and well-being of patients [16]. Similarly, academic dentists have been utilizing ChatGPT to assist in scientific writing [9,11,12]. Examples of its use include translating texts into English [9], employing it as an anti-plagiarism tool, or incorporating it into the theoretical framework [11,12]. However, even the latest versions appear to have limitations in their application [17]. Given the critical nature of accurate information dissemination in healthcare, it becomes imperative to rigorously assess the efficacy of ChatGPT 3.5 in the dental context.

Therefore, the objective of this study was to evaluate the accuracy and completeness of the answers generated by an artificial intelligence website, ChatGPT 3.5, in different areas of dentistry in comparison with expert-formulated questions.

## Materials and methods

Study design and setting

This cross-sectional study was led at the School of Dentistry of Ribeirão Preto University of São Paulo (FORP/USP), Brazil, in June 2023. We used an online AI language model - ChatGPT 3.5, a free version at this point - to converse with, and the responses were collected for analysis.

Ethical aspects

This study was approved by the Institutional Research Ethics Committee (CAAE: 69712923.6.0000.5419) and is consonant with the Helsinki Declaration.

Question preparation

**Figure 1:**
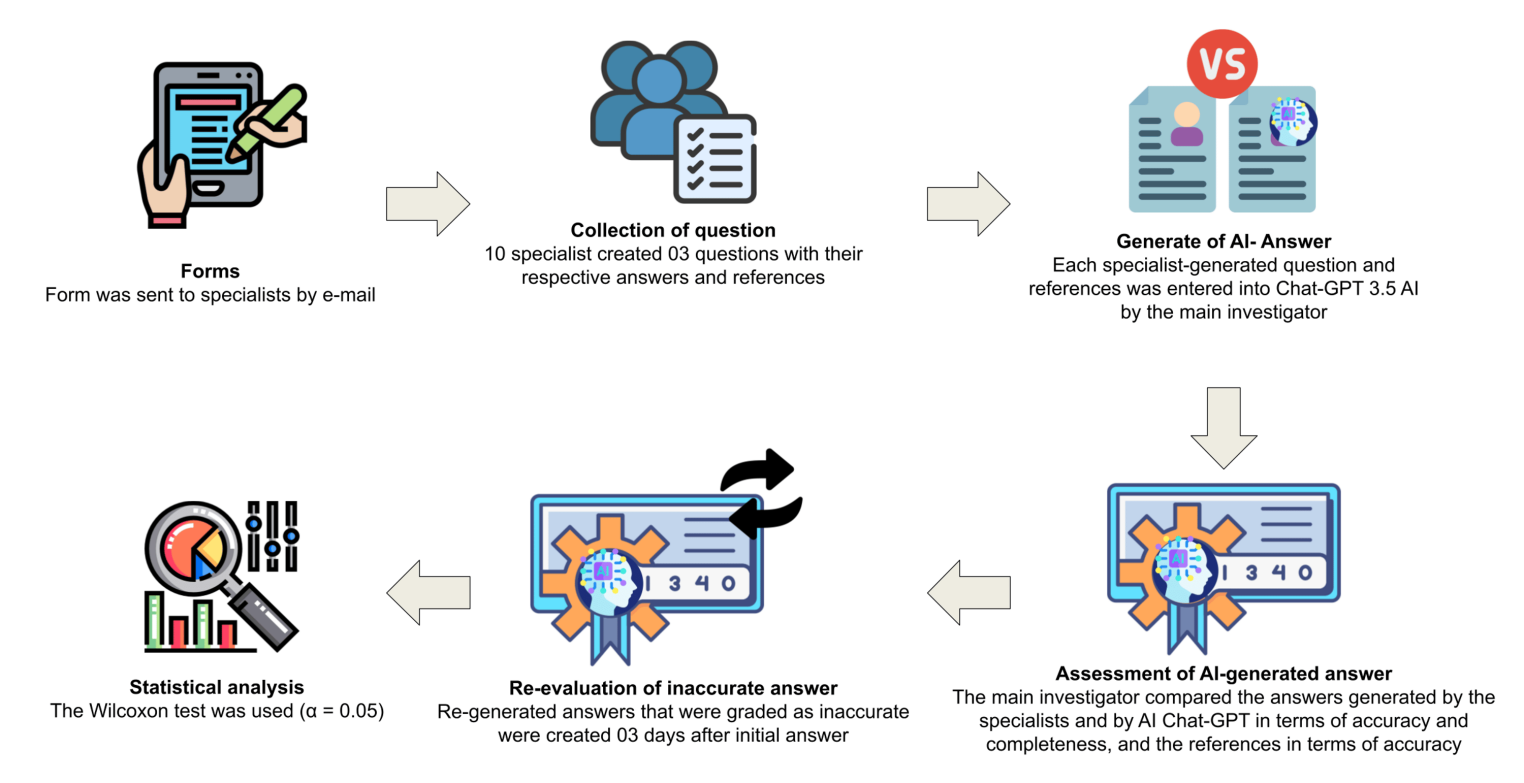
Methodology used in this study. Image Credits: Kelly Fernanda Molena, Author.

Initially, all faculties/experts at FORP/USP were invited via institutional email to read and sign the Free and Informed Consent Form. After that, through a form created by Google Forms, participants were directed to an online page where it was possible to include general information and specialties. Then, they formulated three answers from a clinical or theoretical doubt in the respective area of specialty, the following answer for that question, and bibliographic references through articles or books for that specific answer. A flowchart for an overview of the methods used is presented in Figure 1.

Participants were instructed to provide questions with clear, uncontroversial answers available, dentistry guidance, and unchanged from the beginning of 2021 (counting the training set cut for ChatGPT). These three questions had binary yes/no or right/wrong answers, and the other two were classified as easy, medium, and difficult by subjective classification by the participant/expert who provided the questions and were descriptive or produced a list of multiple correct answers. To minimize bias, participants were asked not to filter questions themselves on ChatGPT.

All questions were subjectively chosen as representative of each participant's specialty. To ensure consistency, all questions were entered into the ChatGPT 3.5 engine by the main investigator, who prompted the chatbot to be specific and incorporate any dental guidelines and references in the answer, if appropriate (with the phrase "Please be specific and incorporate any applicable dental guidelines, as well as accurate bibliographic references, for the question:"). Also, a new command for references was “What is the bibliographical reference for the subject discussed above?" The questions and commands inserted in ChatGPT were in Brazilian Portuguese. These data are presented in Appendix 1. 

Data collection

The data collection ranged from June 5 to July 5, 2023. The questions were used to converse with ChatGPT 3.5 by a single user. The questions were recorded for further analysis. Inaccurate questions (lower than 1 and 2 on the Likert scale) were re-evaluated after three days. The answer provided by the software was copied into a document in Microsoft Word, and it was saved on the computer and Google Drive® for further analysis.

Scoring of answer

An experienced researcher evaluated the accuracy of responses according to two predefined scales of accuracy and completeness. The accuracy scale was a six-point Likert scale (1: completely incorrect, 2: more incorrect than correct, 3: about equal correct and incorrect, 4: more correct than incorrect, 5: almost all correct, 6: correct). This scale was also used to evaluate the accuracy of references through a question generated after the answer ("What is the bibliographic reference for the subject discussed above?").

The response completeness scale was a three-point Likert scale (1: incomplete, addresses some aspects of the question but significant parts are missing or incomplete; 2: adequate, addresses all aspects of the question and provides the minimum amount of information necessary to be considered complete; 3: comprehensive, addresses all aspects of the issue and provides additional information or context beyond what is expected). Answers that are completely incorrect on the accuracy scale (score 1) were not evaluated for completeness.

To assess the reproducibility of results and assess the effect of time on response accuracy, after three days, an internal validation process was carried out in which ChatGPT was repeated with the same questions that generated responses originally classified as inaccurate (less than 3 on the accuracy scale).

Statistical analysis

Score results were listed descriptively (median, mean, interquartile range, standard deviation) and were compared between groups using the Wilcoxon test using IBM-SPSS statistical software (IBM Corp., Armonk, NY). Reassessed questions were compared using the Wilcoxon signed-rank test. Mann-Whitney test was used to compare descriptive and binary questions and to assess the accuracy of references.

## Results

Ninety-three experts were invited to participate. Ten experts (response rate: 10.75%) signed the consent form and completed all stages of the form sent by email. Of these, they belonged to the areas of pediatric dentistry (n = 6); acupuncture (n = 2); endodontics (n = 1); orthodontics (n = 1); oral biology (n = 1), and radiology and imaging (n = 1) and created questions and respective answers and references according to the specialty area. Two experts shared their respective areas of expertise.

The questions and answers belonged to the areas of the participants' specialties and dealt with theoretical and clinical questions in dentistry. A total of 30 questions were created with 15 binary answers (Yes or No), and 15 were descriptive. 

Across all questions (n = 30), the median accuracy score was 5.50 (almost all correct), with a mean score of 4.17 (more correct than incorrect). The median completeness score was 2.00 (adequate, addresses all aspects of the question, and provides the minimum amount of information necessary to be considered complete), with a mean score of 2.07. Comparing accuracy and completeness for binary and descriptive questions, no significant difference was observed at T0 for accuracy (median 6.00 vs. 4.00; mean 4.33 vs. 4.00; p=0.486). The completeness scores for binary and descriptive questions were similar (median 3.00 vs. 2.00; mean 2.33 vs. 1.80; p=0.098). 

When evaluating the references, no significant difference was found between binary and descriptive questions (median 3.00 vs. 3.00; mean 2.73 vs. 2.60; p=0.775). However, the references presented a median value of 3.00, indicating that it was about equal to correct and incorrect. The results are presented in Table 1.

**Table 1:** The Likert Scale was used to assess the accuracy and completeness of the answers generated by ChatGPT according to 30 expert-formulated questions. The questions were descriptive and binary. Inaccurate questions (t0) were re-evaluated after three days (t3). Desc: descriptive. Bin: binary, t0: initial, t3: after inaccurate questions were re-evaluated, N: number, SD: standard deviation, p-value considered p<0.05 to significant difference (*). "-": Absent data.

		N	Mean	Median	SD	Minimum	Maximum	Percentiles		p-value
								25th	75th	
Accuracy		30	4.17	5.50	2.15	1.00	6.00	2.00	6.00	-
Completeness		30	2.07	2.00	0.86	1.00	3.00	1.00	3.00	-
References		30	2.67	3.00	0.71	2.00	5.00	2.00	3.00	-
Accuracy	Bin	15	4.33	6.00	2.44	1.00	6.00	1.00	6.00	0.486
	Desc	15	4.00	4.00	1.89	1.00	6.00	2.50	6.00	
Completeness	Bin	15	2.33	3.00	0.97	1.00	3.00	1.00	3.00	0.098
	Desc	15	1.80	2.00	0.67	1.00	3.00	1.00	2.00	
References	Bin	15	2.73	3.00	0.79	2.00	5.00	2.00	3.00	0.075
	Desc	15	2.60	3.00	0.63	2.00	4.00	2.00	3.00	
Accuracy	t0	9	1.22	1.00	0.44	1.00	2.00	1.00	1.00	0.042*
	t3	9	3.22	2.00	2.22	1.00	6.00	1.00	5.00	
Completeness	t0	9	1.00	1.00	0.00	1.00	1.00	1.00	1.00	0.011*
	t3	9	1.89	2.00	0.60	1.00	3.00	2.00	2.00	

**Figure 2:**
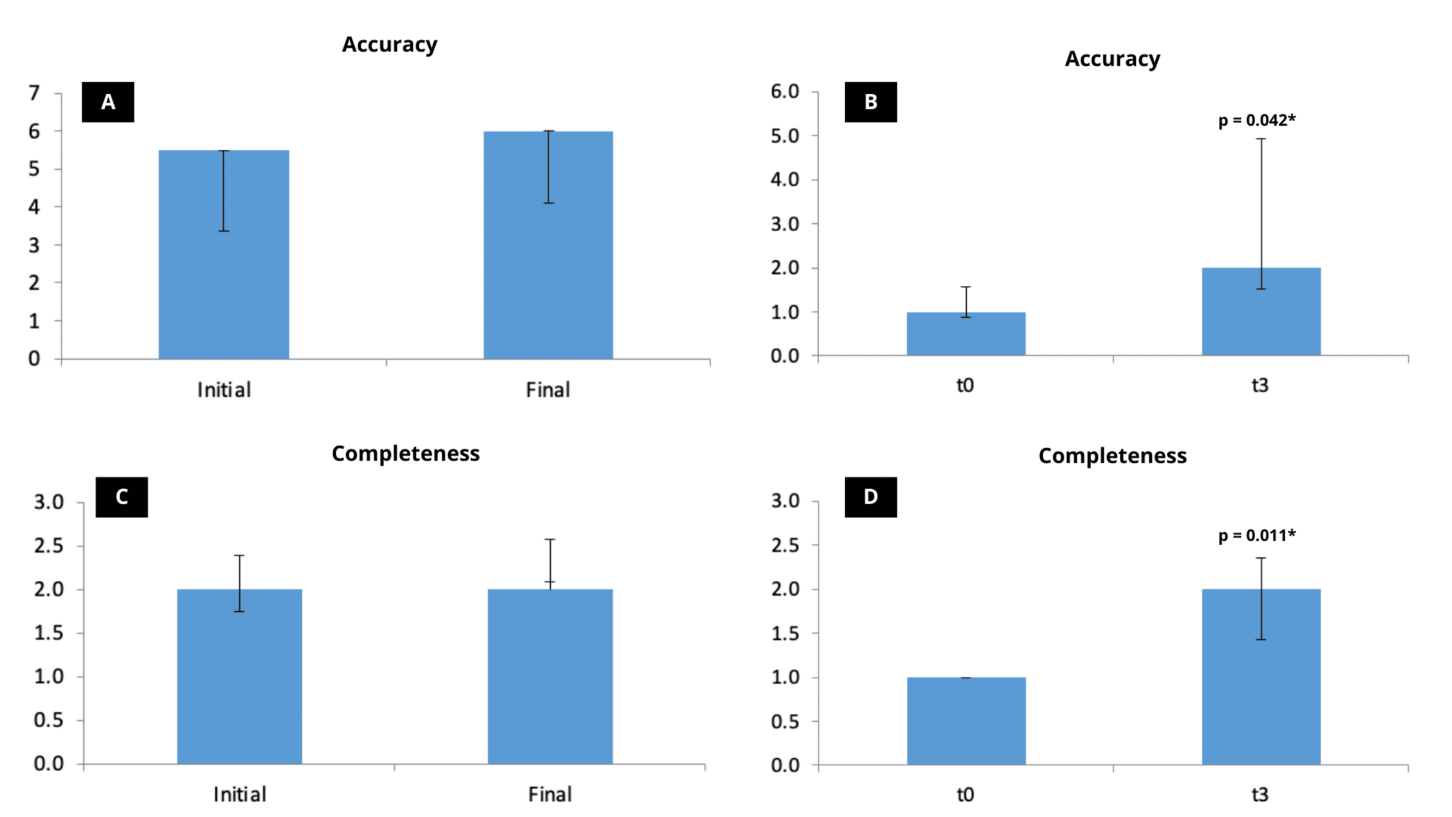
Wilcoxon test was used to evaluate accuracy and completeness of response generated by ChatGPT based on expert-formulated-questions. There were no statistical differences between the initial and final scores for accuracy (A) and completeness (C). However, when re-evaluating imprecise questions (B and D), comparing the initial values of imprecise questions (t0) with those after three days (t3), they were more accurate in t3. *Means statistical difference between the groups.

Nine questions that initially (t0) presented scores 1-2 for accuracy (either incorrect or more incorrect than correct) were re-evaluated after three days (t3). There was a score improvement in accuracy (median 5.50 vs. 6.00; average 4.17 vs. 4.80; p=0.042) and in completeness questions (median 2.00 vs. 2.00; mean 2.07 vs. 2.30; p=0.011). Figure 2 presents these results.

## Discussion

The use of AI in health education and research is increasing [7]. Based on the findings of this study, ChatGPT can partially answer expert-formulated questions with a good level of accuracy and completeness. The responses that were not accurate were replicated, and after three days, they became more accurate and complete, with a significant difference.

The ability to provide improved responses to the same question over time is attributed to machine learning (ML), a subset of AI that enhances its performance through iterative learning from data, as opposed to rule-based approaches in traditional methodologies [18]. Advances in ML have yielded advantages in terms of accuracy, decision-making, rapid processing, cost-effectiveness, and management of intricate data [18,19]. This implies that, despite initially offering partially correct responses to queries, the AI website has refined its response capabilities over time. Consequently, users may find it necessary to pose the same question multiple times to ascertain the accuracy of the response. Nonetheless, despite this iterative improvement, this study still observed instances of incorrect responses. This warrants caution, particularly in healthcare decision-making by professionals or students, as erroneous information could lead to significant harm to individuals [20].

A study by Sallam et al. [21] assessing the technology acceptance model regarding the use of ChatGPT among undergraduate students in health science showed that students perceived the chatbot as having good reliability, validity, and usefulness in the field. Additionally, it can be considered a knowledge transfer tool [22], although some students feel limited in their knowledge when using it [23]. Similarly, several studies evaluated the accuracy of responses generated by ChatGPT in knowledge acquisition [14,15]. These studies found that ChatGPT demonstrates good accuracy in answering questions in various areas, such as microbiology [15] or problem-solving in pathology [14]. Furthermore, it showed potential for knowledge acquisition and clinical problem-solving for medical inquiries [24]. In dentistry, it has been widely used as a diagnostic tool for oral malignancy in radiographs or restorations [2]. Its problem-solving capability may sometimes surpass human capacity in certain scenarios [25]. However, in this study, while ChatGPT was able to answer questions with good accuracy, it could not provide complete answers or correct references.

In the present investigation, ChatGPT demonstrated proficiency in answering easier questions but showed less confidence in responding to those classified as difficult. It appears that ChatGPT performed better when addressing questions related to oral medicine and dentistry categorized as easy or medium difficulty. Questions deemed difficult exhibited lower levels of confidence, particularly those derived from recent research or requiring a high level of expertise for accurate answers. Tasks necessitating critical thinking, reasoning, and interpretation may currently exceed the capabilities of AI systems [11]. Moreover, AI systems like ChatGPT face limitations in handling novel discoveries, complex cases, and intricate reasoning [26].

ChatGPT was not effective at generating references for responses. The references provided were often imprecise or generic. Consequently, questions demanding extensive expertise or sourced from recent scientific literature should be approached cautiously when utilizing this AI tool. Wu and Dang [27] discovered that only 10% of the references generated by ChatGPT were entirely correct. The AI's propensity to fabricate references raises concerns regarding its reliability [28], particularly within the realm of health science [29].

The Likert scale was employed in this study to assess the accuracy and completeness of ChatGPT 3.5’s responses due to its capacity to deliver detailed, consistent, and objective evaluations. This scale enables nuanced differentiation between levels of correctness and is widely used in the literature for similar evaluations [20-22,29]. Its structured approach ensures reliable measurement of AI performance [29] in addressing dental queries.

ChatGPT requires improvements, such as augmenting its database to bolster algorithm training and reduce data bias [4,16]. However, AI can demonstrate prejudice due to its intrinsic design or learning mechanisms, even with representative data devoid of bias [16]. Inadequate or generalized data may result in the creation of incomplete records, potentially leading to misinformation. Particularly within dentistry, this could pose risks to patient health and hinder proper learning for undergraduate and postgraduate students [20]. Continuous refinement in the training and advancement of language models is imperative to enhance their performance and render them suitable for academic applications.

ChatGPT has been utilized for resolving exam questions, exhibiting significant potential for achieving high accuracy, even in medical board examinations [30]. However, it is crucial to adhere to ethics and good practice recommendations when employing AI [16]. Disseminating such knowledge to patients or through scientific publications must be approached with caution to safeguard the health and well-being of individuals [16].

This study has several limitations. First, it utilized a convenience sample and only garnered responses from ten experts, indicating potential bias. Future studies should employ sample calculation techniques and randomization of researchers to enhance validity. Nonetheless, the study holds significance as it addresses a topic impacting educational practices and knowledge acquisition. It specifically evaluates ChatGPT's proficiency in answering dentistry-related queries, but the findings may not apply to other subjects or domains. Additionally, the study only involved interaction with ChatGPT-3.5 by a single user, without comparison to ChatGPT 4.0 or other AI tools. The selection of ChatGPT-3.5 was based on its accessibility, availability as a free tool, and widespread use up to the study's publication. Consequently, conducting a multicentric study in the future could yield more generalizable results.

## Conclusions

ChatGPT partially exhibited good accuracy and completeness and provided incomplete references to solve dentistry questions. When imprecise questions were replicated after a period, they became even more accurate due to ML. AI can be an ally in dentistry for students and researchers, but its use should be approached with caution. The presence of a human with technical training and the ability to critically discern the content is still necessary for complex clinical cases and theoretical knowledge.

Future research should compare ChatGPT 3.5 with other AI models, expand sample sizes, and evaluate real-time clinical applications. Longitudinal and multicentric studies, improved training data, and AI integration in education and decision support systems are also recommended. Additionally, developing ethical guidelines and exploring patient interaction with AI-generated information are essential.

## Appendices

This document has all expert questions, answers, references, and the command given to Open-AI ChatGPT 3.5, between June 5 and July 5, 2023. The original document is in Brazilian Portuguese and was translated to English (https://docs.google.com/document/d/1OM6ho8Msjp1mFj4AwURN6u5FBsvMqZ_YYBGU9DLPEI0/edit?usp=sharing).
